# The effect of the new GMS contract on GP appointment provision in Wales: postal questionnaire survey of practice managers

**DOI:** 10.1186/1756-0500-1-117

**Published:** 2008-11-24

**Authors:** Andrea Edwards, Richard D Neal

**Affiliations:** 1 Victoria Gardens Surgery, Victoria Gardens, Neath, West Glamorgan, SA11 3AY, UK; 2 Department of Primary Care & Public Health, School of Medicine/North Wales Clinical School, Gwenfro Unit 5, Wrexham Technology Park, Wrexham, LL13 7YP, UK

## Abstract

**Background:**

Access to health professionals is a key UK NHS priority, and meeting access targets is rewarded through the new General Medical Services (GMS) contract in the UK. We sought to determine the current state of appointment provision in Wales and any changes resulting from the need to meet indicators in the new GMS contract. We undertook a postal questionnaire study of practice managers in all general practices in Wales.

**Findings:**

Valid responses were received from 396/505 (78.4%) practice managers. 361 (93.1%) practices reported that they had achieved the target for 2004/05. 104 (26%) practices reported that they were 100% open access/advanced access. The most frequent changes reported in response to the new GMS contract were offering more open or advanced access slots (237, 60%), more GP phone consultations (167, 42%), introducing a telephone triage system (100, 25%), introducing a minor illness clinic (76, 19%), and employing or training a nurse practitioner (59, 15%). 83% practice managers believed that patients were able to get an appointment at the time they need it either 'all of the time' or 'most of the time', and 70% that patients were able to get an appointment with the GP of choice either 'all of the time' or 'most of the time'.

**Conclusion:**

This survey has demonstrated the current extent of appointment provision in Wales, and how changes have been driven by incentives. Whether these changes are in the best interests of either patients or doctors, or both, remains to be seen.

## Background

Access to primary care professionals is a key NHS priority,[1] and there is increasing evidence that patients value improved access to primary care.[2] In Wales, an objective for the next decade stated in 2001 was to promote universal and faster access to services,[3] and an important Welsh Assembly Government policy document outlined the following target: 'patients will be able to access an appropriate member of the primary care team within 24 hours of requesting an appointment and much sooner in an emergency'.[4]

Over the past few years, many practices have adopted either a complete open access or advanced access [5,6] model of appointment provision. Open access is usually taken to mean regular surgeries that are not bookable in advance and are often run in combination with booked appointments. Advanced Access is an approach to appointment systems based on measuring and predicting demand each day and matching capacity to demand so that patients can book on the day of their choice.[7] However many practices that purport to use Advanced Access do not use the model's key principles.[8] However there remains contention about the relative advantages and disadvantages, as assessed in different ways from different perspectives, to these systems, especially for certain groups of patients. [9-13] There has been a range of other initiatives aimed at improving access generally. These include: telephone consultations; telephone triage; nurse led minor illness clinics; and the introduction of nurse practitioners and health care assistants, however the evidence for their widespread use remains contentious.

The new General Medical Services (nGMS) contract for general practice was introduced in 2003.[14] The nGMS contract includes financial incentives to practices that achieve an access target; through both the Quality and Outcomes Framework (QoF) (50 bonus points), and the enhanced service category, which differs in each of the four UK countries. Practices in Wales submit an annual plan to their LHB outlining their intention to improve patient access to services. These individual, practice-based plans are then agreed and monitored, and funding of £5000 per average practice size paid. Each LHB has a responsibility to carry out validation checks to ensure that information provided by practices is correct.

We sought to determine the current state of appointment provision in Wales and any changes resulting from the need to meet indicators in the nGMS contract. We were keen to compare the experience in Wales to that of demonstrated by surveys in England and Northern Ireland.[8,15] We undertook this by asking practice managers to answer a brief questionnaire about access in their own practices. We believe that this is of international importance given the variation in the provision of access and in the use of incentives to change practice systems.

## Methods

We conducted a postal questionnaire survey of all practice managers in Wales. We developed and piloted a structured questionnaire that asked questions about the current appointment provision, changes since the nGMC contract, complaints and aggression, their own opinion and their opinion of others' views of the appointment provision, and factual data about the practice. Questions and response options were amended after piloting. Items in the final questionnaire are shown in Table 1.

**Table 1:** Items on questionnaire

Is your practice 100% open/advanced access? *(Y/N)*

If not, in a normal working week, does your practice offer pre-bookable appointments for:
The same day? *(Y/N)*
Next working day? *(Y/N)*
Up to two days or more in advance? *(Y/N)*
Up to one week in advance? *(Y/N)*
Up to one month in advance? *(Y/N)*
More than one month in advance? *(Y/N)*

Has your practice made any of the following significant changes to your appointment system, or the services offered to patients since the introduction of the nGMS contract?
Offered more open/advanced access or less bookable slots? *(Y/N)*
Introduced a telephone triage system for appointments? *(Y/N)*
Employed/trained a nurse practitioner? *(Y/N)*
Introduced a minor illness clinic? *(Y/N)*
More GP phone consultations on a systematic basis? *(Y/N)*

Factual data about the practice:
List size/Number of WTE doctors/nurse practitioners/nurses/healthcare assistants
Did your practice achieve the QoF Access Target 2004/2005? *(Y/N)*

In your opinion, are patients able to obtain an appointment at the time they need it?
*Answer options: all of the time/most of the time/some of the time/not very often, hardly ever/don't know*

In your opinion, do patients get an appointment with the GP of their choice?
*Answer options: all of the time/most of the time/some of the time/not very often/hardly ever/don't know*

In your opinion, what has happened to the number of verbal complaints received at the practice regarding the appointment system?
*Answer options: increased a lot/increased a little/stayed the same/decreased a little/decreased a lot*

In your opinion, what has happened to the level of aggression directed at receptionists if patients don't get the appointment they want when they want it?
*Answer options: increased a lot/increased a little/stayed the same/decreased a little/decreased a lot*

In your opinion, from the perspective of GPs, do you think they consider the current system of appointment provision to be?
*Answer options: very good/good/satisfactory/poor/very poor*

In your opinion, from the perspective of receptionists, do you think they consider the current system of appointment provision to be?
*Answer options: very good/good/satisfactory/poor/very poor*

In your opinion, from the perspective of patients, do you think they consider the current system of appointment provision to be?
*Answer options: very good/good/satisfactory/poor/very poor*

As far as was possible, we used strategies demonstrated in a recent systematic review to maximize response;[16] these included one sheet of double-sided A4 paper only, individualized letters, handwritten envelopes, second class stamped return envelopes, and a reminder to non-responders.

Practice managers were identified from LHB lists and sent the questionnaire with a covering letter and a stamped return envelope in April 2006. Initial non-responders were sent a reminder with a further questionnaire and return envelope one month later.

Data were entered into an Excel spreadsheet. Data entry checks were made on every tenth record. Analysis was undertaken in Excel, and using SPSS. In order to determine whether open/advanced access and QoF target achievement was associated with practice size, t-tests were undertaken.

Given funding and resource limitations we were unable to undertake any validation of the questionnaire responses against other sources of data.

## Results

### Response

From a sample of 505 practice managers, there were a total of 396 (78.4%) valid responses.

### Practice size and personnel

The mean and median numbers of patients per practice were 6459 and 6180. The mean and median numbers of patients per whole-time GP were 1863 and 1857. The mean number of GPs per practice was 3.05. The mean number of nurses, health care assistants and nurse practitioners being 1.68, 0.57 and 0.29. 245 practices employed healthcare assistants ranging from 0.10 – 3.3 WTE. 94 practices employed nurse practitioners ranging from 0.30–3.5 WTE.

### Quality and Outcomes Framework (QoF) access target

361 (93.1%) practices reported that they had achieved the target for 2004/05.

### Access to appointments

104 (26%) practices reported that they were 100% open access/advanced access, with 292 (74%) stating that they were not. Of the practices that were not, 231 (79%) offered 'same day' appointments, 204 (70%) 'the next working day', 206 (70%) 'up to two working days or more in advance', 238 (81%) 'up to one week in advance', 175 (60%) 'up to one month in advance', and 92 (31%) 'more than one month in advance'. These categories were not mutually exclusive with responses covering the range of options that practices were able to offer.

### Significant changes made since nGMS contract

In response to the question about changes made since the nGMS contract, offering more open or advanced access slots was the more frequent response, made by 237 (60%). This was followed by 167 (42%) respondents stating 'more GP phone consultations on a systematic basis', 100 (25%) respondents stating 'introduced a telephone triage system for appointments', 76 (19%) respondents 'introduced a minor illness clinic', and 59 (15%) respondents 'employed/trained a nurse practitioner'.

### Appointments at time of patient need and with doctor of choice

The responses to these two questions are summarized in Table 2. This shows that 83% of practice managers believed that patients were able to get an appointment at the time they need it either 'all of the time' or 'most of the time', and that 70% of practice managers believed patients were able to get an appointment with the GP of choice either 'all of the time' or 'most of the time'.

**Table 2:** Responses to questions about appointments at time of patient need and with doctor of choice

	All of the time (%)	Most of the time (%)	Some of the time (%)	Not very often (%)	Hardly ever (%)	Don't know (%)
Are patients able to obtain an appointment at the time they need it?	59 (15)	271 (68)	63 (16)	3 (1)	0 (0)	0 (0)

Do patients get an appointment with the GP of their choice?	55 (14)	223 (56)	115 (29)	2 (1)	1 (0)	0 (0)

### Verbal complaints and aggression

The responses to these two questions are summarized in Table 3. This shows the biggest response category for both questions was 'stayed the same', with 45% answering this for verbal complaints and 47% for aggression towards receptionists. There were slightly more responses for the 'decreased' options for both questions than the 'increased' options.

**Table 3:** Responses to the questions about verbal complaints and aggression

	Increased a lot (%)	Increased a little (%)	Stayed the same (%)	Decreased a little (%)	Decreased a lot (%)	Don't know (%)
What has happened to the number of verbal complaints received at the practice regarding the appointment system?	11 (3)	69 (17)	179 (45)	71 (18)	65 (17)	1(0.2)
What has happened to the level of aggression directed at receptionists if patients don't get the appointment they want when they want it?	21 (5)	79 (20)	187 (47)	58 (15)	49 (13)	2 (0.5)

### Practice managers' views of how others regard the appointment system

The last question asked practice managers, in their opinion, what other groups considered the current system of appointment provision to be like. These groups were GPs, receptionists, and patients. The findings are summarized in Table 4. This shows that whilst all three groups were perceived to rate their systems highly, there were differences between them, with patients' views, and to a lesser extent receptionists' views were perceived slightly less positive than GPs' views.

**Table 4:** Practice managers' views of how others regard the appointment system

Practice managers' view of how others regard their current system of appointment provision	Very good (%)	Good (%)	Satisfactory (%)	Poor (%)	Very poor (%)
GP perspective	136 (34)	164 (41)	80 (20)	16 (4)	0 (0)
Receptionist perspective	125 (31)	140 (35)	92 (23)	37 (9)	2 (1)
Patient perspective	82 (21)	164 (41)	121 (31)	27 (7)	2 (1)

### Association of open or advanced access and QoF target achievement with practice size

Open or advanced access was associated with smaller practice list size (mean difference 901, t = -2.32, 95%CI -1666, -137, p = 0.02). QoF target achievement was not associated with practice list size (mean difference 1212, t = -1.78, 95% CI -129, -2552, p = 0.08), although a much larger sample size would be needed to detect this.

## Discussion

### Summary of main findings

One quarter of practices were 100% open or advanced access. Of the others, four fifths offered same day appointments, three fifths up to one week in advance, and one third more than a month in advance. The commonest changes to appointment provision reported were more open access slots, and more systematic use of GP telephone consultations. A large majority of practice managers believed that their current systems enable patients to get an appointment at the time they need it, and with their GP of choice. Practice managers reported no changes in either verbal complaints or aggression relating to appointment provision. They perceived that GPs, receptionists and patients all regarded their appointment systems positively. Open or advanced access was associated with smaller practice list size.

### Strengths and weaknesses

For a postal questionnaire study we achieved an excellent response rate, which was greater than the English survey (245/391, 63%), [8] but lower than the Northern Irish survey (94%), although this only had 59 respondents.[15] Contributory factors to this may have included: a topical and relevant subject, a short and well-designed questionnaire; the fact that practice managers are rarely consulted in research studies of this nature; and the questionnaire coming from a fellow practice manager. The list size per practice and per GP are in keeping with other data from Wales, and the sample responding to the questionnaire was similar in these respects to practices in Wales overall. Data from the MDSi Contract Manager database  reported that 92.8% of practices in Wales achieved the access bonus. This figure is very close to the data reported here, again suggesting that the sample responding to the questionnaire was similar in these respects to practices in Wales overall. The weaknesses are those of any questionnaire: we were limited to fixed response options, and had to assume a certain truth in the responses, taking the results at face value. We also cannot be sure that there is no systematic bias from the non-responders. Lastly, this was only a survey of practice managers and was not therefore able to address the view of patients or other health professionals. Their view may differ considerably. One of our questions (question 2), was, in retrospect, slightly ambiguous, and the findings from this question must be interpreted with additional caution. Additionally the wording of 'open' and 'advanced' access may have caused some confusion.

### Conclusion/discussion of the findings within the context of the literature

This questionnaire survey has demonstrated the current extent of appointment provision in general practices in Wales and changes that have occurred since the implementation of the nGMS contract. The contract has led to a move towards more open/advanced access appointments and more telephone consulting. Whilst this may help practices achieve QoF access targets, its effect on the overall quality provision of general practice remains unknown. The effect of incentives on changing appointment provision has been demonstrated, and is similar to the findings from Northern Ireland.[15] The level of access to appointments in general practices in Wales, and the views of practice managers have been demonstrated. There is an issue about who access best serves: the patient or the practice, and the trade off between faster access and quality of care (e.g. longer consultations and reduced continuity of care).[12,13] There is also an issue about whether patients actually get appointments when they need rather than want them, and whether they are with their doctor of choice. For example working patients and those with complex chronic conditions may express a clear preference for pre-bookable appointments rather than faster access. This issue has not been resolved by the nGMS contract. It may be that some practices are choosing not to fully achieve the access target because they feel it is neither in their, nor their patients' best interests.

## Competing interests

The authors declare that they have no competing interests.

## Authors' contributions

The original idea for the study came from AE. RDN and AE jointly designed the study. AE undertook the fieldwork and conducted the analysis. RDN wrote the first draft of the paper. Both authors contributed to the final draft of the paper.

## References

[B1] Department of Health (2000). The NHS Plan. A plan for investment, a plan for reform.

[B2] Welsh Assembly Government, 2003 (2003). The future of primary care: an action plan for primary care in Wales.

[B3] National Assembly for Wales (2001). Improving health in Wales – a plan for the NHS and its partners.

[B4] Welsh Assembly Government (2002). The future of primary care – an action plan for primary care in Wales.

[B5] Murray M, Tantau C (1998). Must patients wait?. Joint Commission Journal on Quality Improvement.

[B6] Warrender TS (2002). Promoting advanced access in primary care.

[B7] Murray M, Berwick DM (2003). Advanced Access. Reducing waiting and delays in primary care. JAMA.

[B8] Goodall S, Montgomery A, Banks J, Salisbury C, Sampson F, Pickin M (2006). Implementation of Advanced Access in general practice: postal survey of practices. British Journal of General Practice.

[B9] White S, Jones M (2004). Impact of advanced access. British Journal of General Practice.

[B10] Pascoe SW, Neal RD, Allgar VL (2004). Open access versus bookable appointment systems: survey of patients attending appointments with general practitioners. Br J Gen Pract.

[B11] Ahluwalia S, Offredy M (2005). A qualitative study of the impact of the implementation of advanced access in primary healthcare on the working lives of general practice staff. BMC Fam Pract.

[B12] Salisbury C, Goodall S, Montgomery AA, Pickin DM, Edwards S, Sampson F (2007). Does Advanced Access improve access to primary health care? Questionnaire survey of patients. British Journal of General Practice.

[B13] Salisbury C, Montgomery AA, Simons L, Sampson F, Edwards S, Baxter H (2007). Impact of Advanced Access on access, workload, and continuity: controlled before-and-after and simulated-patient study. British Journal of General Practice.

[B14] Department of Health (2003). The new GMS contract 2003: investing in general practice.

[B15] Meade JG, Brown JS (2006). Improving access for patients – a practice manager questionnaire. BMC Family Practice.

[B16] Edwards P, Roberts I, Clarke M, DiGuiseppi C, Pratap S, Wentz R, Kwan I, Cooper R (2003). Methods to increase response rates to postal questionnaires. The Cochrane Database of Methodology Reviews 4.

